# Evaluating the Prevalence and Clinical Significance of Fetal Posterior Cerebral Artery Variants via Magnetic Resonance Imaging: Insights From a Tertiary Healthcare Facility in Tamil Nadu

**DOI:** 10.7759/cureus.64128

**Published:** 2024-07-09

**Authors:** Dhivya Gunasekaran, Karthik Krishna Ramakrishnan, Ajina Sam, Karpagam R K, Paarthipan Natarajan

**Affiliations:** 1 Radiodiagnosis, Saveetha Medical College and Hospital, Saveetha Institute of Medical and Technical Sciences, Saveetha University, Chennai, IND

**Keywords:** mra, cva, anatomy of circle of willis, mr imaging, fetal pca

## Abstract

Introduction

The prevalence and clinical significance of fetal posterior cerebral artery (FPCA) variants are studied using magnetic resonance imaging (MRI) at Saveetha Medical College. This research focuses on the fetal origin of the posterior cerebral artery (PCA), a condition where the posterior communicating artery (PComA) is larger than the P1 segment of the PCA, affecting cerebral hemodynamics and associated with various cerebrovascular pathologies.

Materials and methods

This retrospective analysis employed MRI records from the Department of Radio Diagnosis at Saveetha Medical College, conducted between January 2013 and December 2023. The study included patients undergoing MRI for various neurological indications, with specific imaging protocols including T1- and T2-weighted sequences, diffusion-weighted imaging, and magnetic resonance angiography (MRA).

Results

The study confirmed a prevalence rate of FPCA variants within the expected range of 20%-30%. MRI findings were systematically analyzed by experienced radiologists to assess the presence and characteristics of FPCA variants. The relationship between these variants and clinical symptoms was explored, revealing significant correlations that emphasize the variants' impact on patient outcomes, particularly in the context of cerebrovascular events.

Conclusion

The study underlines the importance of recognizing FPCA variants due to their significant implications in neurovascular health and cerebrovascular accident (CVA) outcomes. These variants alter cerebral hemodynamics and can complicate neurosurgical and diagnostic procedures. Therefore, personalized treatment and management strategies are crucial for optimizing patient care.

## Introduction

The posterior cerebral artery (PCA) plays a vital role in the brain's vascular system, delivering blood to key regions such as the occipital lobes, the inferior temporal lobes, and the thalamus. Variations in PCA anatomy, particularly the fetal origin of the PCA, are of significant clinical interest. The term "fetal PCA" describes a condition where the posterior communicating artery (PComA) is larger than the P1 segment of the PCA. This anatomical variation can influence cerebral blood flow and has been linked to various cerebrovascular disorders [1].

Fetal posterior cerebral artery (FPCA) variants are relatively common, occurring in approximately 20%-30% of the population [2]. These variants are marked by the predominance of the PComA in supplying the PCA territory, effectively incorporating part of the posterior circulation into the anterior circulation. This unique arrangement can impact collateral circulation patterns, potentially affecting clinical outcomes in cerebrovascular events such as strokes [3].

Understanding these anatomical variants is not only of scholastic interest but also holds substantial clinical importance, especially in diagnostic radiology and neurosurgery. Magnetic resonance angiography (MRA) utilized in our study at a tertiary healthcare center in Chennai provides a non-invasive and highly accurate method to assess these vascular variations. This technique offers valuable insights into cerebral vascular architecture without the need for contrast agents or invasive procedures [4,5,6].

In our retrospective study conducted at a tertiary healthcare center in Chennai, we used MRI to evaluate the prevalence of FPCA variants and their clinical implications. This analysis not only maps the regional prevalence of these variants but also enhances our understanding of their impact on patient outcomes, particularly in the context of cerebrovascular accidents (CVA).

## Materials and methods

This retrospective cross-sectional study utilized magnetic resonance imaging (MRI) brain scans and MRA data collected over a 10-year period, from January 2013 to December 2023, at a tertiary healthcare facility in South India. The purpose was to examine the prevalence and characteristics of FPCA configurations among a large, diverse sample of patients. The design allowed for a comprehensive analysis of historical imaging data to identify trends and patterns in the anatomical variation of the Circle of Willis. Ethical approval was granted under SRB number 186/04/2024.

The study encompassed a total of 12500 MRI and MRA scans from patients who visited the healthcare center for various neurological evaluations. The population included both genders, across a wide age range of adults who received care at this high-volume center, making it a representative sample of the broader South Indian population. This extensive dataset provides a robust foundation for evaluating the prevalence of FPCA and its distribution across different demographic segments within the region.

Inclusion criteria encompassed individuals aged 18 years or older with high-quality MRI and MRA scans that clearly depict the Circle of Willis, who underwent imaging as part of standard diagnostic care without any specific selection for research purposes initially.

Exclusion criteria included patients with any history of cerebral surgeries potentially altering the anatomical structure of the Circle of Willis, MRI scans that were incomplete or of poor quality impairing accurate assessment of vascular anatomy, and patients with pathological conditions that could distort the normal anatomy of the Circle of Willis, such as tumors or vascular malformations. Patient data, comprising age, gender, clinical symptoms, and specific MRI findings, were documented using a standardized data collection form. Descriptive statistics were employed to summarize demographic and clinical characteristics. Utilizing Chi-square tests for categorical data with a significance level set at p<0.05, the study examined the association between the presence of FPCA variants and clinical symptoms. This methodology was selected to ascertain whether statistically significant associations existed between PCA variants and clinical outcomes.

MRA offers non-invasive vascular assessment without ionizing radiation or contrast agents. Techniques include non-contrast magnetic resonance angiography (NCMRA), comprising black and bright blood imaging. NCMRA methods, such as time of flight (TOF) and phase contrast (PC) MRA, are complemented by emerging approaches like steady-state free precession (SSFP)-based and electrocardiogram (ECG)-gated fast spin echo (FSE) MRA. TOF-MRA utilizes short repetition time (TR) gradient echo sequences to enhance signal contrast, with 2D TOF suitable for slower-flowing vessels and 3D TOF for high resolution. Challenges include flow saturation and sensitivity to short T1 tissues in TOF MRA. Data reformation employs maximum intensity projection (MIP) or volume rendered technique (VRT) to enhance vessel visualization and necessitate source image review for accurate assessment.

The Circle of Willis is a crucial circulatory anastomosis encircling the optic chiasma and hypothalamus ensuring a network of blood supply to the brain and maintaining blood flow balance between anterior and posterior regions. It forms where the terminal branches of the vertebral and internal carotid arteries converge. Its main components include the anterior cerebral arteries, terminal branches of the internal carotid arteries supplying the frontal and superior medial parietal lobes, and the PCAs, terminal branches of the basilar artery supplying the occipital and inferomedial temporal lobes. Additionally, it is completed by two connecting vessels, the anterior communicating artery facilitating arterial supply crossover between left and right anterior cerebral arteries and the PComA, linking the internal carotid artery to the PCA, vital for collateral circulation.

The cerebrum's blood supply primarily comes from three cerebral arteries: the anterior cerebral arteries nourish the anteromedial surfaces of the frontal and superior medial parietal lobes, the middle cerebral arteries supply the outer convexity of the cerebral hemispheres, and the PCAs provide for the medial and lateral parts of the occipital lobes and the inferomedial temporal lobes. Originating from the basilar artery, the PCA divides into several segments: the P1 segment begins at the end of the basilar artery and extends to the PComA; the P2 segment subdivides into anterior and posterior sub-segments as it encircles the midbrain; the P3 segment courses through the quadrigeminal cistern to the sulci of the occipital lobe; and the P4 segment lies within the calcarine fissure of the occipital lobe. Finally, the P5 segment includes crucial branches like the calcarine and parieto-occipital arteries, which are essential for visual processing.

## Results

**Figure 1:**
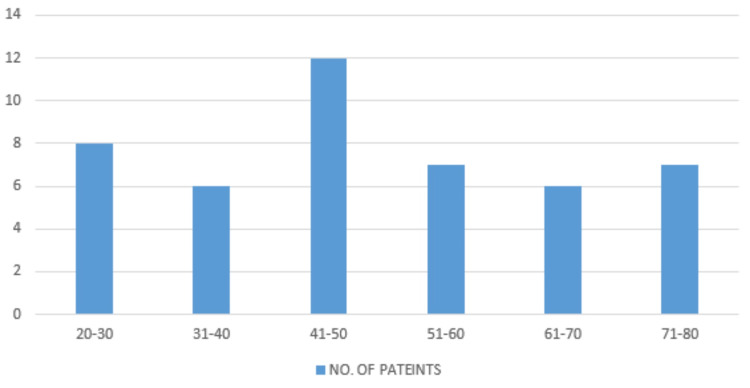
Age distribution of FPCA The bar chart represents the age distribution of patients who underwent MRI scans. The data is represented as the number of patients (N) in each age group. The age groups are categorized into 20-30, 31-40, 41-50, 51-60, 61-70, and 71-80 years. Statistical significance is considered at p<0.05 and p<0.001. FPCA: fetal posterior cerebral artery; MRI: magnetic resonance imaging

The bar chart (Figure 1) illustrating patient age distribution reveals a notable concentration in the 41-50 and 51-60 age groups, indicating a higher frequency of MRI scans among middle-aged individuals. This trend likely reflects an increased prevalence of neurological symptoms or conditions requiring diagnostic imaging in these age brackets. The elevated incidence within these ranges may signal the onset or peak of cerebrovascular and neurodegenerative diseases, which are more common during middle age. This observation highlights the importance of targeted screening and diagnostic measures to address the specific healthcare needs of this demographic, facilitating early detection and intervention in neurological disorders.

**Figure 2:**
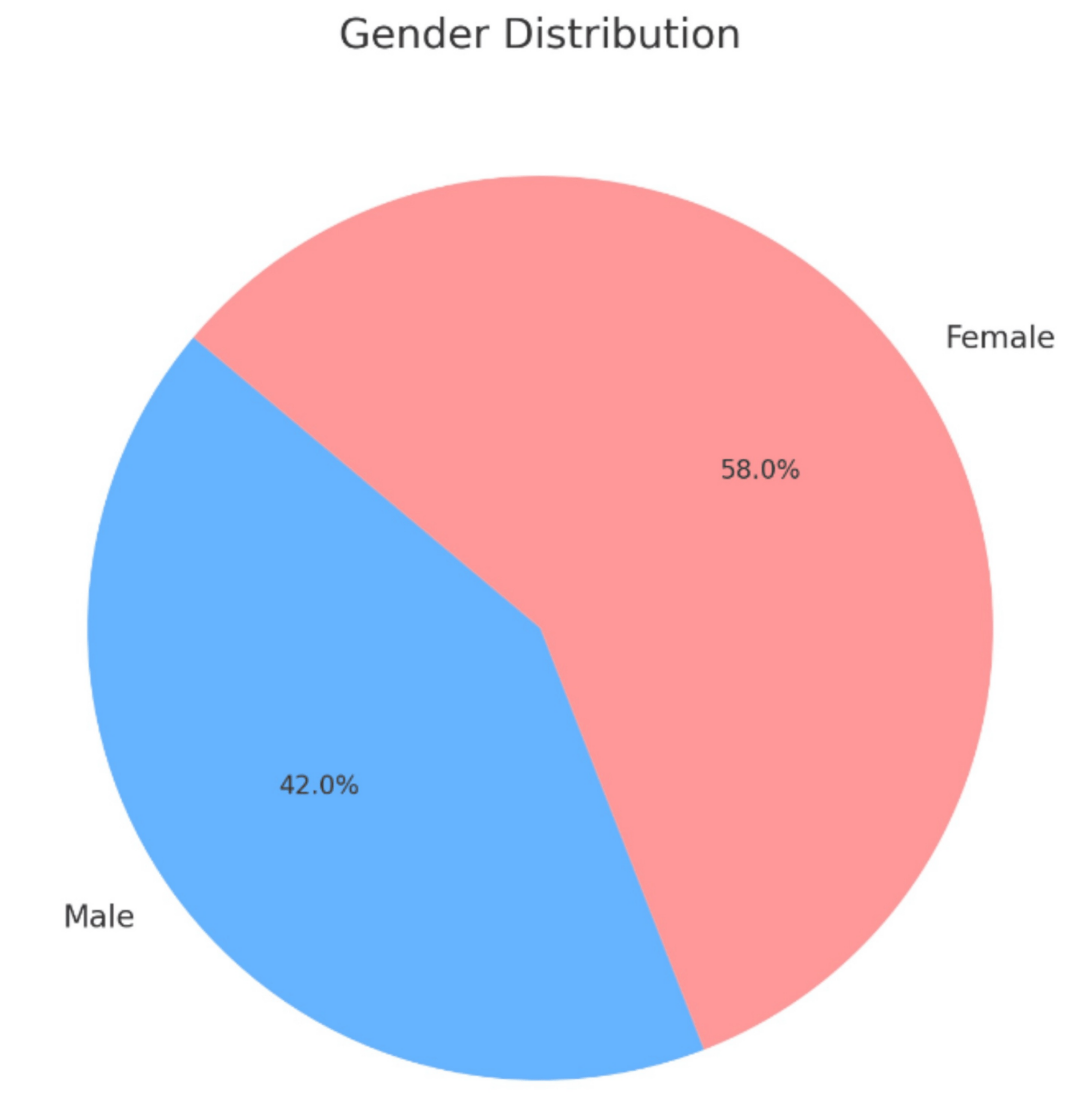
Gender distribution The pie chart represents the gender distribution of patients who underwent MRI scans. The data is represented as the percentage (%) of male and female patients within the study population. Female patients constitute 58.0% of the sample, while male patients constitute 42.0%. Statistical significance is considered at p<0.05 and p<0.001. MRI: magnetic resonance imaging

The pie chart (Figure 2) illustrating patient gender distribution shows a slight predominance of female patients 7250 (58%) compared to male patients 5250 (42%). This gender imbalance suggests potential underlying factors influencing healthcare utilization patterns or disease prevalence within the population. It is plausible that certain neurological conditions, such as migraines or multiple sclerosis, exhibit differing prevalence rates between genders, contributing to the observed disparity in MRI scan frequencies.

**Figure 3:**
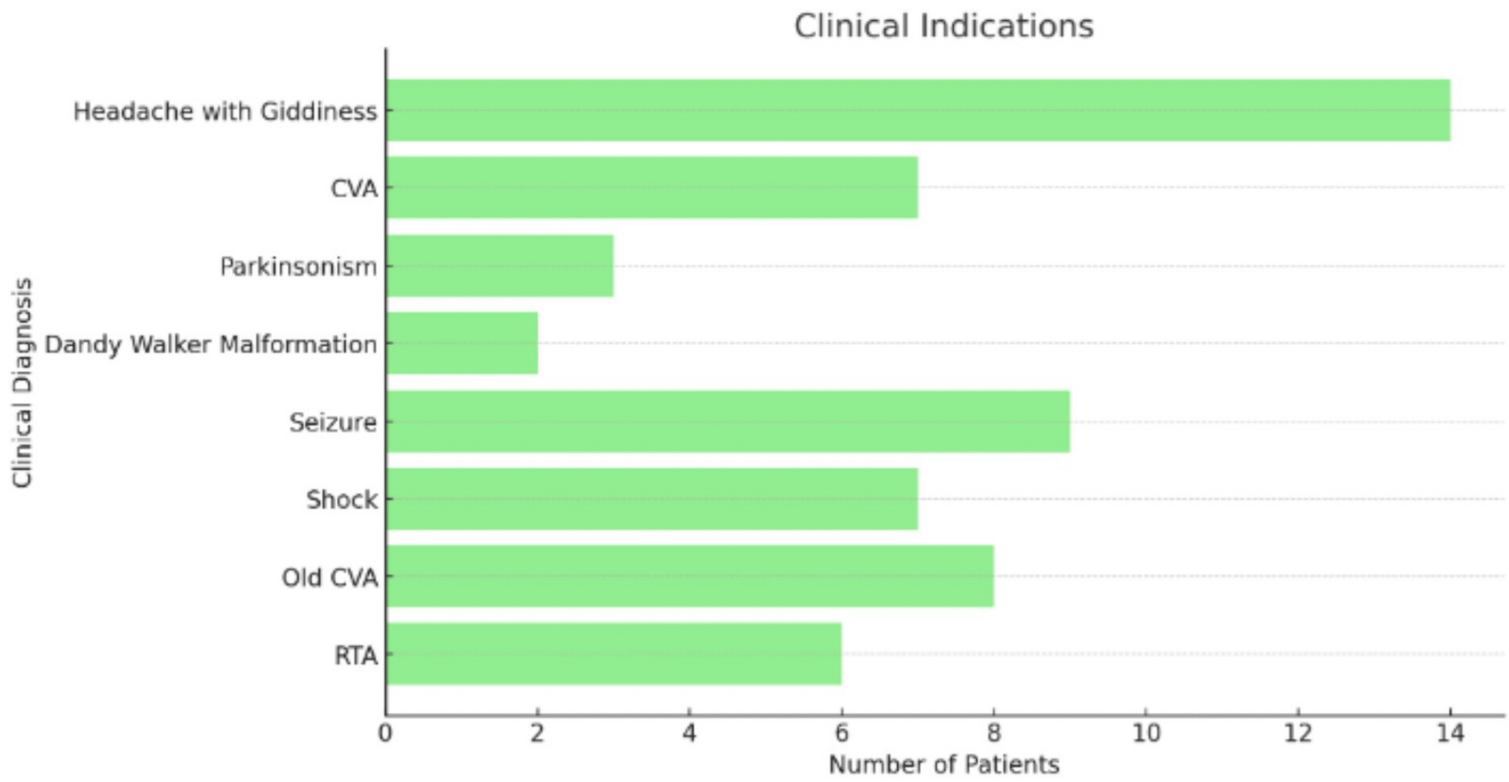
Clinical indications The horizontal bar chart represents the clinical indications for MRI scans among the study population. The data is represented as the number of patients (N) for each clinical diagnosis. The clinical diagnoses include headache with giddiness, CVA, parkinsonism, Dandy-Walker malformation, seizure, shock, old CVA, and RTA. Statistical significance is considered at p<0.05 and p<0.001. MRI: magnetic resonance imaging; RTA: road traffic accident; CVA: cerebrovascular accident

The horizontal bar chart (Figure 3) depicting clinical indications highlights the pivotal role of MRI scans in diagnosing brain-related conditions, with "Headache with giddiness" and "Cerebrovascular Accident (CVA)" emerging as the most common reasons for imaging. This prevalence underscores the urgency and complexity of cases presenting with acute and chronic neurological symptoms. Headaches accompanied by dizziness can signal a range of underlying issues, from benign vertigo to potentially life-threatening conditions such as tumors or stroke. Similarly, CVA requires immediate imaging to determine the type and extent of the stroke, facilitating timely interventions critical for patient outcomes. The prominence of these indications underscores the indispensable role of MRI scans in the comprehensive evaluation and management of neurological disorders.

**Figure 4:**
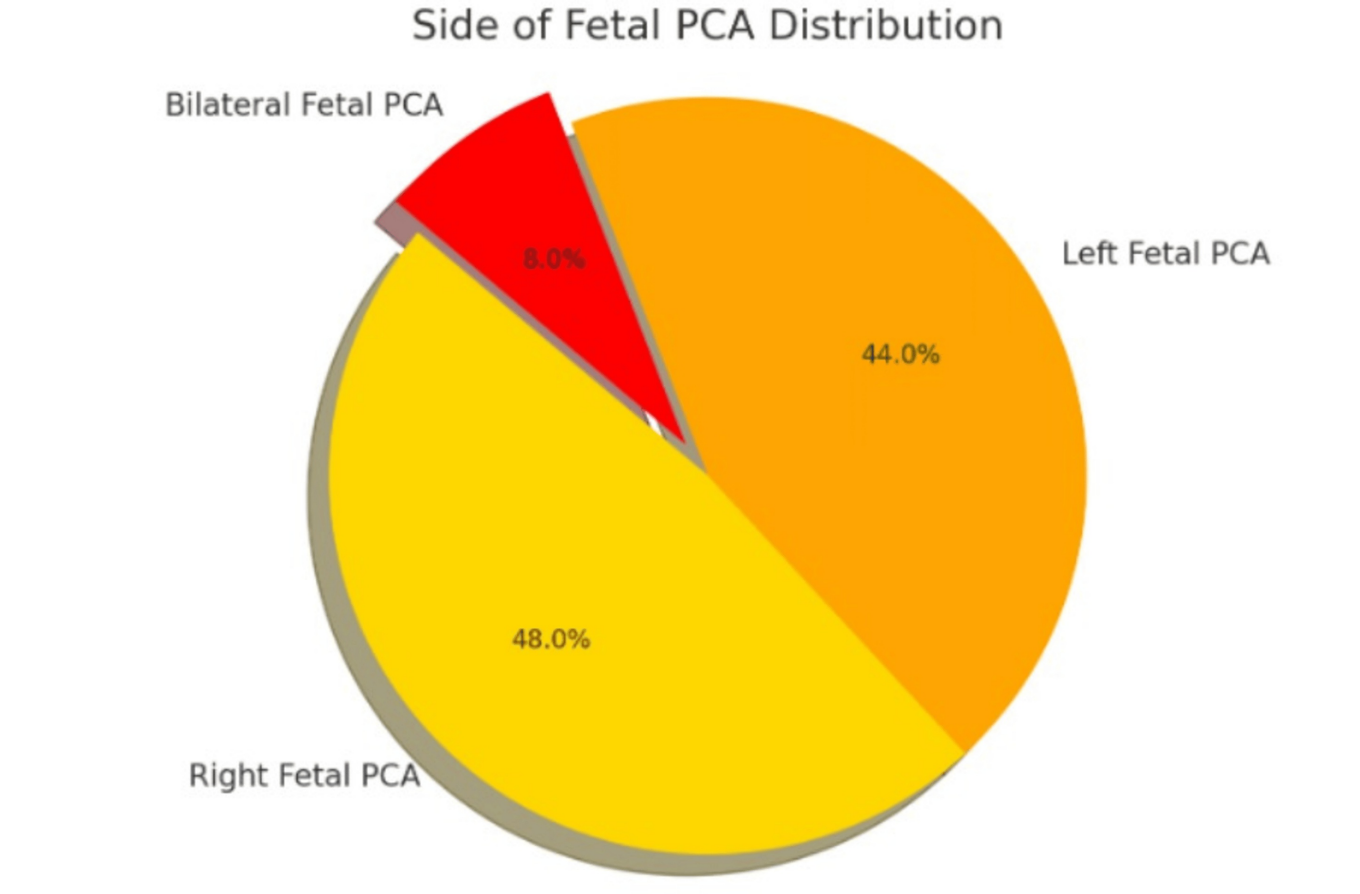
Side of FPCA distribution The pie chart represents the distribution of the side of FPCA among the study population. The data is represented as the percentage (%) of patients with left FPCA, right FPCA, and bilateral FPCA. Specifically, 5500 (44.0%) of patients have left FPCA, 6000 (48.0%) have right FPCA, and 1000 (8.0%) have bilateral FPCA. Statistical significance is considered at p<0.05 and p<0.001. FPCA: fetal posterior cerebral artery

**Figure 5:**
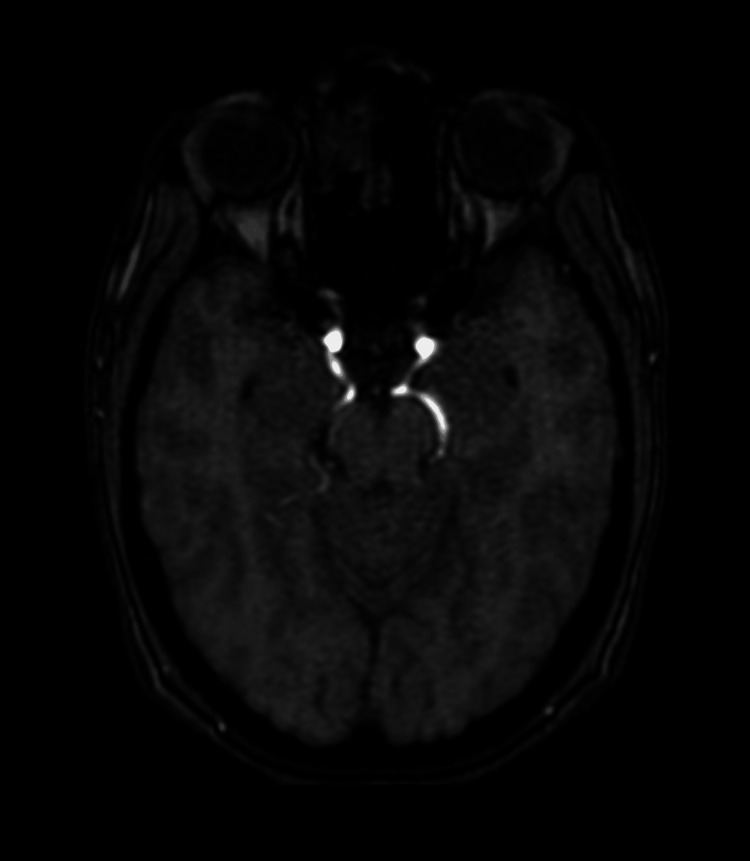
MRA image showing bilateral FPCA MRA: magnetic resonance angiography; FPCA: fetal posterior cerebral artery

**Figure 6:**
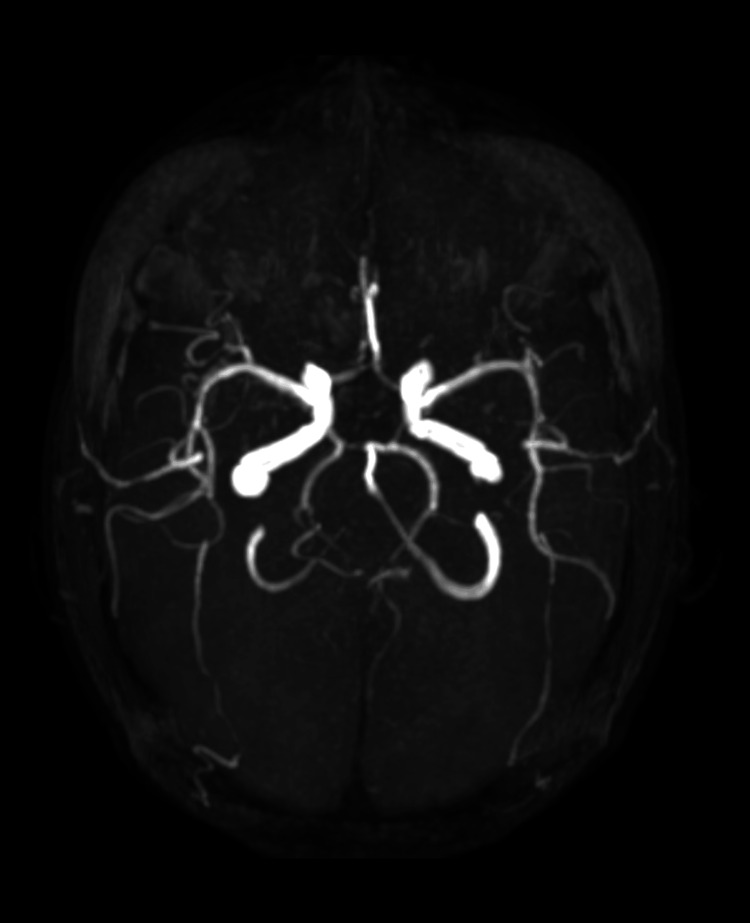
MIP image showing bilateral FPCA MIP: maximum intensity projection; FPCA: fetal posterior cerebral artery

**Figure 7:**
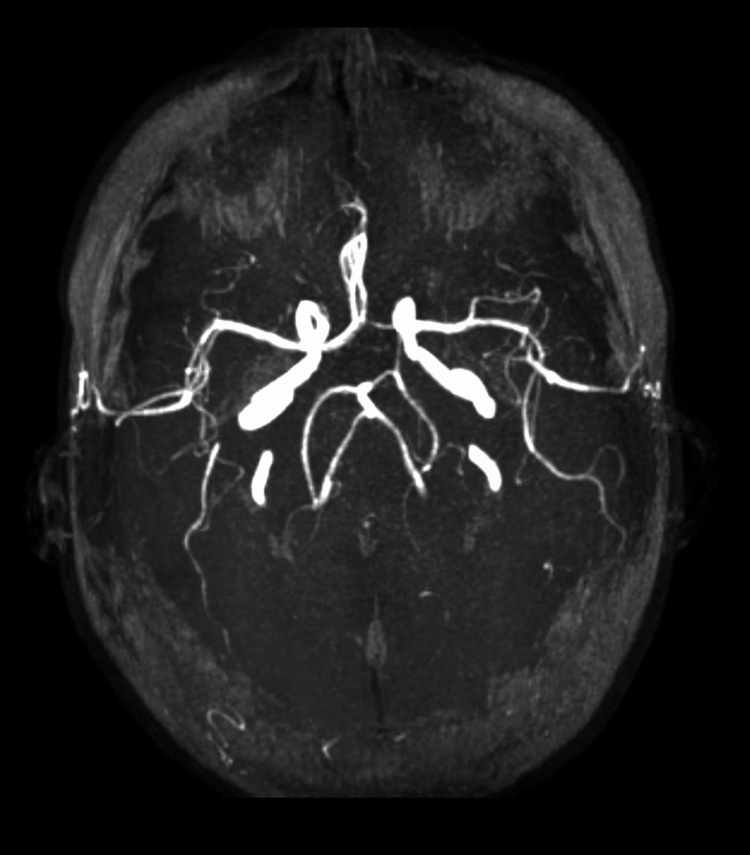
MIP showing left FPCA FPCA: fetal posterior cerebral artery; MIP: maximum intensity projection

**Figure 8:**
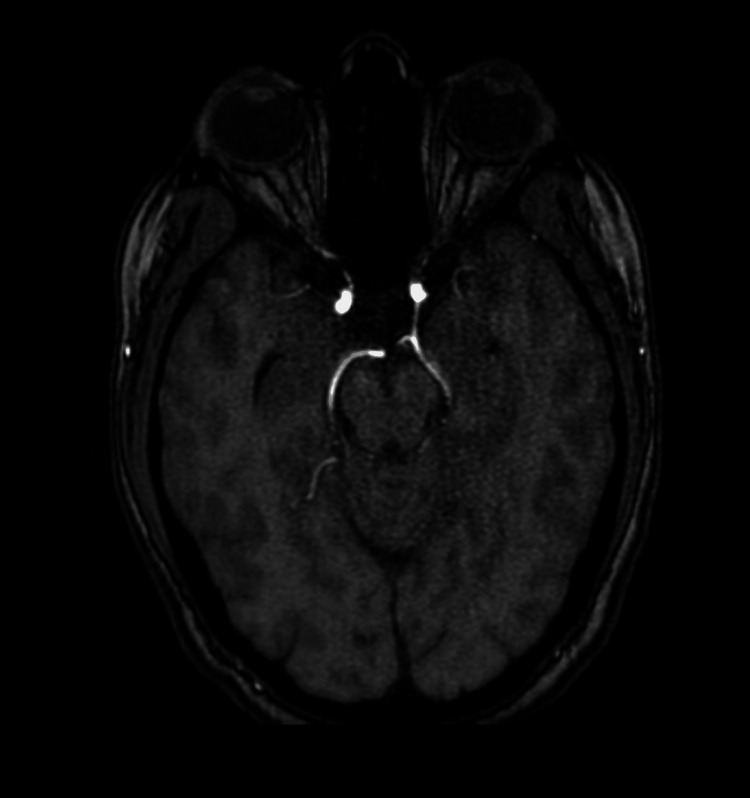
MRA showing left FPCA MRA: magnetic resonance angiography; FPCA: fetal posterior cerebral artery

**Figure 9:**
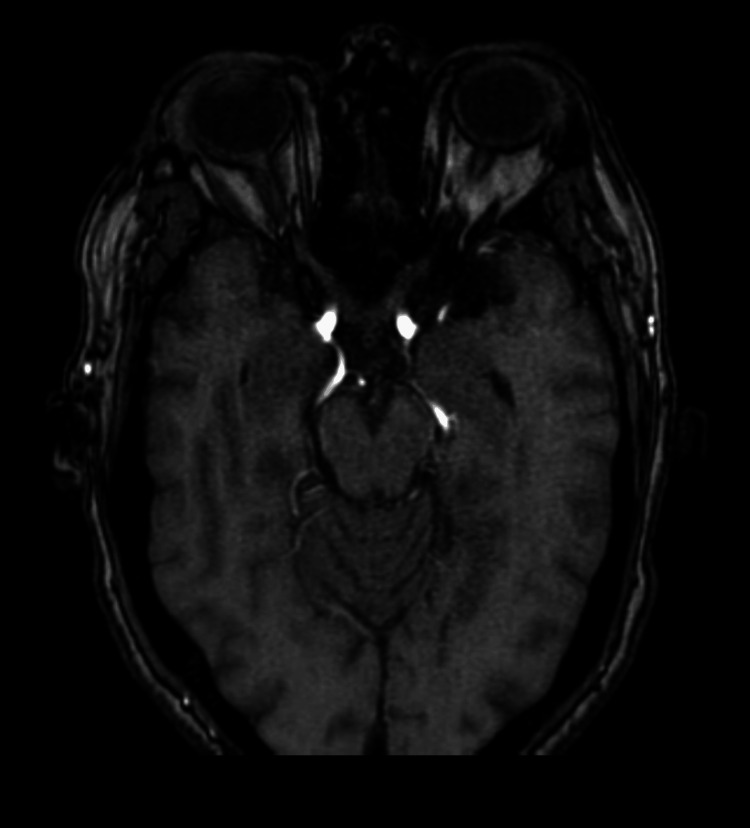
MRA showing right FPCA FPCA: fetal posterior cerebral artery; MRA: magnetic resonance angiography

**Figure 10:**
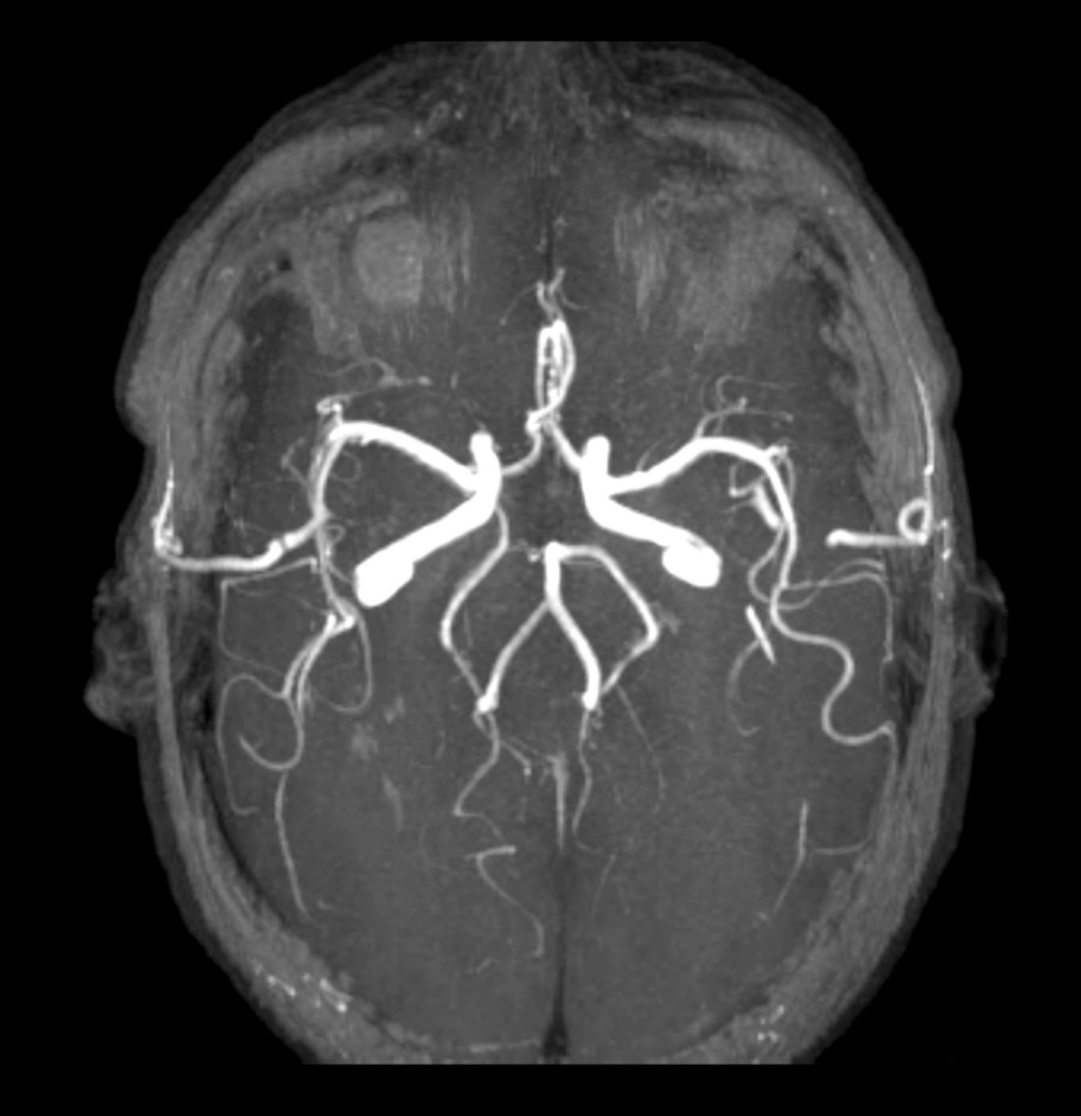
MIP showing right FPCA MIP: maximum intensity projection; FPCA: fetal posterior cerebral artery

The pie chart (Figure 4) depicting the distribution of FPCA side reveals a notable prevalence of either right or left FPCA, with a smaller proportion exhibiting bilateral involvement. This distribution holds significant clinical implications as the side of the FPCA can influence stroke symptoms and associated risks for each patient. Unilateral FPCA involvement may disrupt the balance of blood flow in the brain, potentially heightening the risk of ischemic events in specific cerebral territories due to asymmetrical perfusion. Conversely, bilateral FPCA, although less common, may signify a more evenly distributed flow pattern albeit still atypical, which could influence the patient's susceptibility to certain neurological conditions. Understanding this distribution aids in tailoring interventions and treatment strategies to address the unique cerebrovascular dynamics of each patient, thereby optimizing clinical outcomes and minimizing the risk of adverse events (Figures 5-10).

The presence of a complete FPCA variant, where the internal carotid artery supplies the PCA solely via the PComA due to the underdevelopment or absence of the P1 segment from the basilar artery, holds significant clinical implications. Individuals with this variant face heightened vulnerability to ischemic events affecting the internal carotid artery as their posterior cerebral regions depend entirely on this artery for blood supply. 

The partial FPCA variant, characterized by a dual blood supply from both the internal carotid artery through the PComA and the basilar artery via a smaller-than-usual P1 segment, holds significant clinical implications. This variant offers a protective collateral pathway, augmenting cerebral blood flow and potentially mitigating the impact of arterial obstructions by sustaining perfusion to critical brain regions. Recognition of a partial FPCA is paramount in stroke management, as it informs accurate diagnosis and treatment planning, particularly in determining the source of ischemia and devising reperfusion strategies. Advanced imaging techniques such as MRA or computed tomography angiography (CTA) play a crucial role in precise identification, enabling clinicians to assess vascular configurations and tailor surgical or interventional approaches accordingly, thereby optimizing patient outcomes and minimizing risks associated with cerebrovascular events (Table 1).

**Table 1:** Types of FPCA Statistical significance is considered at p<0.05 and p<0.001. FPCA: fetal posterior cerebral artery

FPCA type	Male distribution	Female distribution	Total individuals
Complete FPCA	820 (50.82%)	793 (49.18%)	1613 (57.15%)
Partial FPCA	605 (50%)	605 (50%)	1210 (42.85%)

## Discussion

The demographic data showing a higher number of female patients (58%) than male patients (42%) aligns with global health observations that women are more likely to seek medical care than men. Additionally, women have a higher prevalence of certain neurological disorders such as migraines and multiple sclerosis, which might necessitate more frequent use of diagnostic imaging tools like MRI. Understanding these gender disparities is crucial for developing targeted health policies and prevention strategies to address specific needs effectively.

In the study population, young adults aged between 20 and 30 years represent a significant demographic, comprising 18% of the total. Within this age group, there is growing concern regarding neurological issues, which present unique challenges given their impact on individuals during their prime working years. These concerns underscore the necessity for proactive measures such as early detection programs aimed at mitigating the potential long-term disabilities associated with these neurological conditions. By identifying and addressing these issues early on, it becomes possible to enhance the overall well-being and productivity of this vital segment of the population and ensure they can lead fulfilling and productive lives without being hindered by preventable neurological ailments [7].

Among the study participants, middle-aged adults, aged 41 to 50 years, constitute the largest demographic, comprising 24% of the total population. This age range often marks a critical period where the initial symptoms of degenerative diseases, such as Parkinson's disease, or early signs of cerebrovascular issues may emerge. The prevalence of such conditions underscores the significance of routine health screenings within this age group. Early detection through regular screenings can facilitate timely intervention and management, potentially mitigating the progression of these diseases and improving long-term outcomes. By prioritizing proactive healthcare measures, individuals in this demographic can better safeguard their neurological health and overall well-being, ensuring a higher quality of life as they navigate through this pivotal stage of adulthood [8].

In elderly adults aged 61-80 years, who make up 28% of the population, there is an elevated risk of diseases such as stroke, Alzheimer’s, and other forms of dementia. This highlights the critical need for advanced diagnostic tools to effectively manage and treat these age-related neurological conditions. Implementing such tools could significantly enhance the quality of life for this demographic by enabling earlier detection and more targeted interventions [9].

Headaches and giddiness, often challenging to diagnose due to their nonspecific nature, require sophisticated imaging to rule out serious underlying causes, such as tumors or vascular anomalies, and to guide appropriate treatment. The presence of patients with both recent and old CVAs indicates the crucial role of MRI in assessing the extent of brain damage, planning rehabilitation, and preventing future strokes. The diverse neurological presentations, ranging from seizures and Parkinsonism to shock and road traffic accidents, underscore MRI's versatility in diagnosing and managing a wide array of acute and chronic neurological diseases. Variants of FPCA have significant clinical implications, influencing cerebral hemodynamics and affecting the risk and severity of cerebral ischemic events. Understanding PCA anatomy is crucial for accurately predicting areas at risk during cerebrovascular insults and for planning surgical interventions. Tailored patient management, particularly in stroke care, can benefit from knowledge of specific PCA anatomy, optimizing reperfusion strategies, and preventive measures. MRA plays a vital role in modern neurology by providing a non-invasive method to visualize cerebral blood vessels without contrast agents, which is particularly beneficial for patients with renal impairment or allergies to contrast media. The study’s findings demonstrate MRA's high diagnostic yield, with 70% of scans revealing significant abnormalities, highlighting its clinical value.

The literature on PCA and its variants, particularly fetal-type PCA, reveals a complex interplay of anatomical diversity and clinical implications, significantly influencing neurovascular pathology and therapeutic strategies. Studies consistently report a prevalence of fetal-type PCA in approximately 20%-30% of the population, highlighting its relevance in cerebrovascular incidents. These anatomical variants, characterized by an alternative blood supply route via the internal carotid artery rather than the basilar artery, predispose individuals to unique stroke patterns and complicate neurosurgical interventions. Advances in diagnostic imaging, such as MRA, have improved the detection and understanding of these variants, enabling tailored management approaches in stroke care. Research underscores the necessity for meticulous vascular assessment to optimize patient outcomes, particularly in acute stroke management where PCA variants may alter expected ischemic patterns. The ongoing exploration of PCA variants aims to refine diagnostic protocols and therapeutic interventions, ensuring that neurovascular treatments align closely with individual anatomical profiles, thereby enhancing both prognostic predictions and clinical outcomes as can be seen in Table 2.

**Table 2:** Review of literature PCA: posterior cerebral artery; CT: computed tomography; PComA: posterior communicating artery; MCA: middle cerebral artery

Authors (citation number)	Year	Title	Key findings
Chaves C, et al. [1]	2012	Posterior cerebral artery	Detailed review of PCA-related stroke syndromes
Lambert SL, et al. [2]	2004	Neuroangiographic anatomy and common cerebrovascular diseases	Discusses neuroangiographic anatomy in clinical practice
Alpers BJ, et al. [3]	1959	Anatomical studies of the Circle of Willis in normal brain	Provides foundational anatomical details of the Circle of Willis
Saeki N, et al. [4]	1977	Microsurgical anatomy of the upper basilar artery and the posterior Circle of Willis	Explores detailed microsurgical anatomy relevant to neurosurgery
Zeal AA, et al. [5]	1978	Microsurgical anatomy of the posterior cerebral artery	Focuses on the microsurgical aspects of the PCA
Van der Lugt A, et al. [6]	2004	Accuracy of CT angiography in the assessment of a fetal origin of the posterior cerebral artery	Evaluates the diagnostic accuracy of CT angiography for PCA variants
Jongen JC, et al. [7]	2002	Blood supply of the posterior cerebral artery by the carotid system on angiograms	Studies the carotid contributions to PCA blood supply
Pedroza A, et al. [8]	1987	Microanatomy of the posterior communicating artery	Details the microanatomy of the PComA and its clinical significance
Bulsara KR, et al. [9]	2007	Anatomic variant of the posterior cerebral artery	Discusses variations in PCA anatomy and their implications
Kolukısa M, et al. [10]	2015	Carotid endarterectomy in a patient with posterior cerebral artery infarction: influence of fetal type PCA on atypical clinical course	Case study on PCA infarction and surgical intervention
Senol MG, et al. [11]	2009	Simultaneous posterior and middle cerebral artery infarct	Examines cases with simultaneous PCA and MCA infarcts
Kovač JD, et al. [12]	2014	Intracranial arterial variations: a comprehensive evaluation using CT angiography	Comprehensive assessment of intracranial arterial variations
Horikoshi T, et al. [13]	2002	Magnetic resonance angiographic evidence of sex-linked variations in the Circle of Willis and the occurrence of cerebral aneurysms	Examines sex-linked variations in the Circle of Willis and related aneurysms
Kayembe KN, et al. [14]	1984	Cerebral aneurysms and variations in the Circle of Willis	Studies the relationship between Circle of Willis variations and cerebral aneurysms

The clinical significance of the FPCA lies in its impact on cerebrovascular dynamics and stroke patterns. Individuals with this variant often have different ischemic stroke presentations due to the unique collateral circulation provided by the PComA. The literature describes increased susceptibility to certain CVAs and aneurysms in the presence of an FPCA. The variant affects hemodynamic stability and can lead to specific vulnerabilities in the vascular structure, influencing both acute and chronic neurological outcomes.

MRA is pivotal in diagnosing PCA variants. It offers a non-invasive approach with high-resolution images, which is crucial for detailed vascular mapping without exposing patients to ionizing radiation or contrast-induced nephropathy. The research underscores the necessity of integrating targeted MRA sequences into routine brain imaging protocols to ensure accurate identification of PCA variants, thereby facilitating appropriate clinical management.

Ongoing research is focusing on the embryological development of PCA variants to better understand their origins and potential links to neurodevelopmental outcomes. This research is anticipated to lead to earlier and more precise diagnoses, allowing for interventions that could mitigate the lifelong impacts of these anomalies. Furthermore, advancements in genetic screening may soon provide insights into the predisposition to these vascular variations, offering new avenues for prevention and personalized medicine. Capone and colleagues' recent work provides a deeper understanding of the developmental anomalies associated with FPCA, which could lead to improved diagnostic and intervention strategies at earlier developmental stages, potentially improving neurodevelopmental outcomes.

The unique challenges posed by FPCA variants require customized management strategies. These include the use of tailored surgical approaches to avoid disrupting critical vascular structures and targeted pharmacotherapy to manage the risk of stroke associated with these anomalies [10-14]. Additionally, the development of guidelines for managing patients with these variants is crucial for improving clinical outcomes. The necessity for customized therapeutic approaches is highlighted by Lambert et al., who discuss the need for tailored intervention strategies based on detailed vascular anatomy. Such personalized treatment plans are vital for optimizing patient outcomes and mitigating risks associated with abnormal vascular structure.

This comprehensive review underscores the complexity of PCA variants and their profound implications for neurovascular health. The variability in their presentation and impact highlights the need for advanced diagnostic techniques and individualized management strategies. Continued research and collaboration across specialties are essential to refine our understanding and improve patient care and outcomes in neurovascular health.

Despite the valuable insights gained from studying FPCA variants via MRI, several limitations are acknowledged. The study's sample size and the specific demographic characteristics may limit the generalizability of the findings, necessitating larger, more diverse populations to confirm results and ensure applicability across different ethnic and age groups. MRI's inherent limitations in resolution and potential imaging artifacts can affect the accuracy of detecting and characterizing vascular anomalies. Additionally, functional MRI studies have shown that FPCA variants can influence vascular responses due to altered cerebral autoregulation, introducing systematic confounds in interpreting functional connectivity and neural activation patterns. The clinical significance of these anatomical variations remains an area requiring further investigation to better understand their contribution to neurological symptoms and outcomes. Variations in MRI technology and imaging protocols also pose challenges, emphasizing the need for standardization across studies to ensure data comparability and reproducibility. Furthermore, understanding the genetic and developmental underpinnings of PCA variants is still in its early stages, highlighting the need for future research focused on embryological development and genetic predispositions to these vascular anomalies.

## Conclusions

This retrospective analysis conducted at a tertiary health care hospital, examining 12500 patients, provided significant insights into the utilization of MRI and MRA across diverse demographics and neurological indications. The study revealed notable gender and age disparities, with a predominance of female patients and a significant representation of middle-aged and elderly individuals, highlighting the need for targeted healthcare strategies. Tailored healthcare policies could enhance access to neurological diagnostics for women, who are at greater risk for conditions like multiple sclerosis and migraines, and address age-related disorders such as Alzheimer’s disease and stroke through preventive screenings and early diagnostic interventions. The study also underscored the versatility and indispensability of MRI in diagnosing complex neurological conditions, with clinical indications ranging from headaches and giddiness to CVAs and seizures. These findings validate the critical role of advanced neuroimaging in modern medical diagnostics, providing essential insights that guide patient management and treatment strategies effectively.
